# Clinical Application of Artificial Ascites in assisting CT-guided Percutaneous Cryoablation of Hepatic Tumors Adjacent to the Gastrointestinal Tract

**DOI:** 10.1038/s41598-017-17023-8

**Published:** 2017-11-30

**Authors:** Bing Li, Chuan Liu, Xiao-Xue Xu, Yang Li, Yong Du, Chuan Zhang, Hou-Jun Zheng, Han-Feng Yang

**Affiliations:** Sichuan Key Laboratory of Medical Imaging, Department of Radiology, Affiliated Hospital of North Sichuan Medical College, Sichuan Province, Nanchong, 637000 China

## Abstract

This study was to assess the safety and efficacy of artificial ascitetes in assisting CT-guided cryoablation of hepatic tumors adjacent to the gastrointestinal (GI) tract. A total of 84 patients with peripheral hepatic tumors adjacent to the GI tract, who were treated cryoablation, were included in this retrospective study. Of these 84 patients, cryoablation had been performed in 39 patients with 41 peripheral hepatic tumors. These were assisted by induction artificial ascites while 40 patients with 43 peripheral hepatic tumors underwent cryoablation without induction of ascites. The artificial ascites separation success rate, the cryoablation technique effectiveness, local tumor progression and complications were all evaluated. The results showed that the artificial ascites separation success rate for 41 hepatic tumors adjacent to the GI tract was 95% (39/41). Technique effectiveness of group I was achieved in 35 of 43 tumors (81.3%) after follow-up imaging three months after cryoablation. In group II, technique effectiveness was achieved in 39 of 41 tumors after follow-up imaging three months following cryoablation. No major complications were encountered in either of the two groups. Artificial ascites assisting in CT-guided percutaneous cryoablation is a reliable and effective method for the treatment of hepatic tumors adjacent to the GI tract, and it can achieve a fine local control of such tumors.

## Introduction

Cryoablation technique destroys targeted tissue conferring low temperature to it^1^. It induces protein denaturation, cellular dehydration, and microcirculatory failure during the repeated freezing and thawing process. Cryoablation has the advantage of being able to be monitored intra procedurally, as there is clear visualization of the entire ice ball with computed tomography (CT) imaging^2^. This has been successfully carried out for treating primary and metastatic hepatic tumors patients in patients where surgical resection is out of equation^1,2^.

Cryoablation of tumors adjacent to GI tract can damage these organs as ice ball of more than 3.0 mm to that of the tumor margin is required to cause complete cell death^3^. Moreover, there are several reports in literature suggesting increase usage of cryoablation in such cases could also damage the adjacent organs like the stomach, colon etc. In addition to these issues, incidence of GI perforation (0.08–0.70%, as per several reports) has made safe and effective implementation of percutaneous cryoablation to hepatic tumors adjacent to the GI tract a challenge for clinicians. Recently, many reports suggest artificial induction of ascites to protect the organs adjacent to the tumors from injury during such ablation treatments^4–6^.

Patel *et al*.^3^ successfully implemented cryoablation, with induction of artificial ascites, to treat renal tumors adjacent to the GI tract without any damage to adjacent organs or related complications. Furthermore, several studies have reported that hepatic tumors adjacent to the GI tract could be treated safely and effectively with radio frequency (RF) and microwave (MW) ablation after induction of an artificial ascites^7–10^. To our knowledge, though, cryoablation has not been used to treat hepatic tumors abutting the GI tract so far. We have undertaken this retrospective study to assess the safety and efficacy of CT-guided percutaneous cryoablation assisted by artificial ascites for treatment of such tumors.

## Results

### Patient population

Patient demographics characteristics and tumor characteristics in the two groups are summarized in Table 1. There was no significant differences in mean age, gender, or tumor size between the two groups. Tumors in the Group - I trended to be smaller than Group - II (2.6 cm vs. 2.8 cm, p > 0.05), but were not statistical significant (Table 1).

**Table 1:** Patient, tumor, and procedure characteristics.

	Ablation without ascites group-I (n = 43)	Ablation with ascites group-II (n = 41)	*p*
Patient characteristics			
Age (in years) mean ± SD (range)	61 ± 8.7	62.5 ± 9.1	> 0.05
Gender (male/female)	26/14	24/15	> 0.05
Tumor characteristics			
Hepatocellular carcinoma	26	22	
Hepatic metastases	16	17	
Hepatic adenoma	1	2	
tumor size (in cm) mean ± SD (range)	2.6 ± 1.2 (1.3–4)	2.8 ± 0.9 (1.1–4)	> 0.05
Adjacent to gastrointestinal tract			
Stomach	12	16	
Small or large intestine	31	25	
Ablation procedure characteristics			
Maximum diameter of the ablation zone (in cm) mean ± SD (range)	4.4 ± 1.6 (3.3–5.2)	4.8 ± 2.1 (3.3–6.3)	> 0.05
Ablation time (min)	12 ± 6 (12–24)	12 ± 8 (12–24)	
Technical effectiveness (3 months follow-up)	35/43 (81.3%)	39/41 (95%)	< 0.05
Complications	13	6	< 0.05
Gastrointestinal tract injury	6	0	
Gastrointestinal perforation	0	0	
Low-grade Fever	5	4	
Pleural effusion	2	2	

### Artificial ascites

Artificial ascites induction was achieved successfully in all patients. The time required for induction of sufficient artificial ascites ranged from 7.0 to 16.0 min (mean = 9 min). The mean volume of 2.0% iodinated contrast, which was injected into peritoneal cavity, was 460.0 ± 280.0 ml (range 300.0–1,000.0 ml) (Table 2). The artificial ascites separation success rate of 41 hepatic tumors adjacent to the GI tract was 95% (39/41). The displacement between tumor and adjacent GI tract was 1.3 ± 0.7 cm. In two patients, obvious adhesion was still observed between the stomach and the liver surface on CT after injecting artificial ascites; One patient with a previous laparotomy two years ago and one patient had undergone transcatheter hepatic arterial chemo-embolization (TACE) treatment before cryoablation. As confirmed by ultrasound, the infused artificial ascites in all patients disappeared spontaneously within four days. The characteristics of artificial ascites procedure are summarized in Table 2.

**Table 2:** Artificial ascites procedure characteristics.

Separation success rate	39/41 (95%)
Mediums of artificial ascites	2% iodinated contrast
Volume of Artificial ascites (ml) mean ± SD (range)	460 ± 280.6 (300–1000)
Displacement of adjacent gastrointestinal tract (cm) mean ± SD (range)	1.3 ± 0.7 (0.5–2.2)

### Outcome of cryoablation

**Figure 1:**
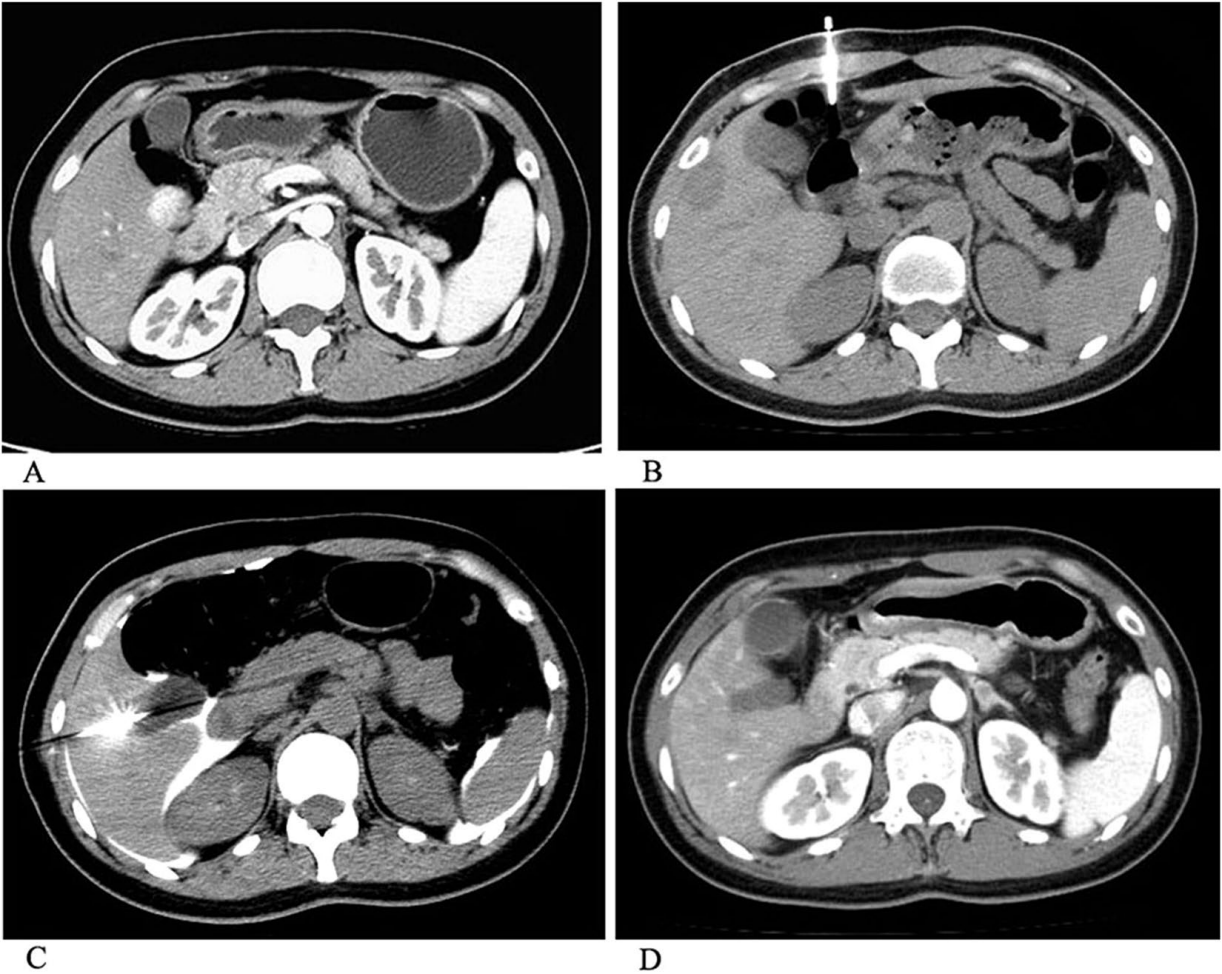
Artificial ascites assisting percutaneous cryoablation of a hepatic adenoma adjacent to the duodenum in a 34 year old female. (**A**) CT demonstrates a hepatic adenoma adjacent to the duodenum. (**B**) A 20-gauge introducer needle was inserted into the peritoneal cavity along the edge of the liver under CT guidance. (**C**) Then, a sufficient amount of 2.0% iodinated contrast was injected; we see the artificial ascites separates the tumor and adjacent duodenum. Two freeze/thaw cycles (12.0 min freeze, 3.0 min passive thaw) were applied. CT demonstrates the ice ball covering the tumor completely. (**D**) Contrast-enhanced CT showed tumor was complete ablation after 3 months followed up.

**Figure 2:**
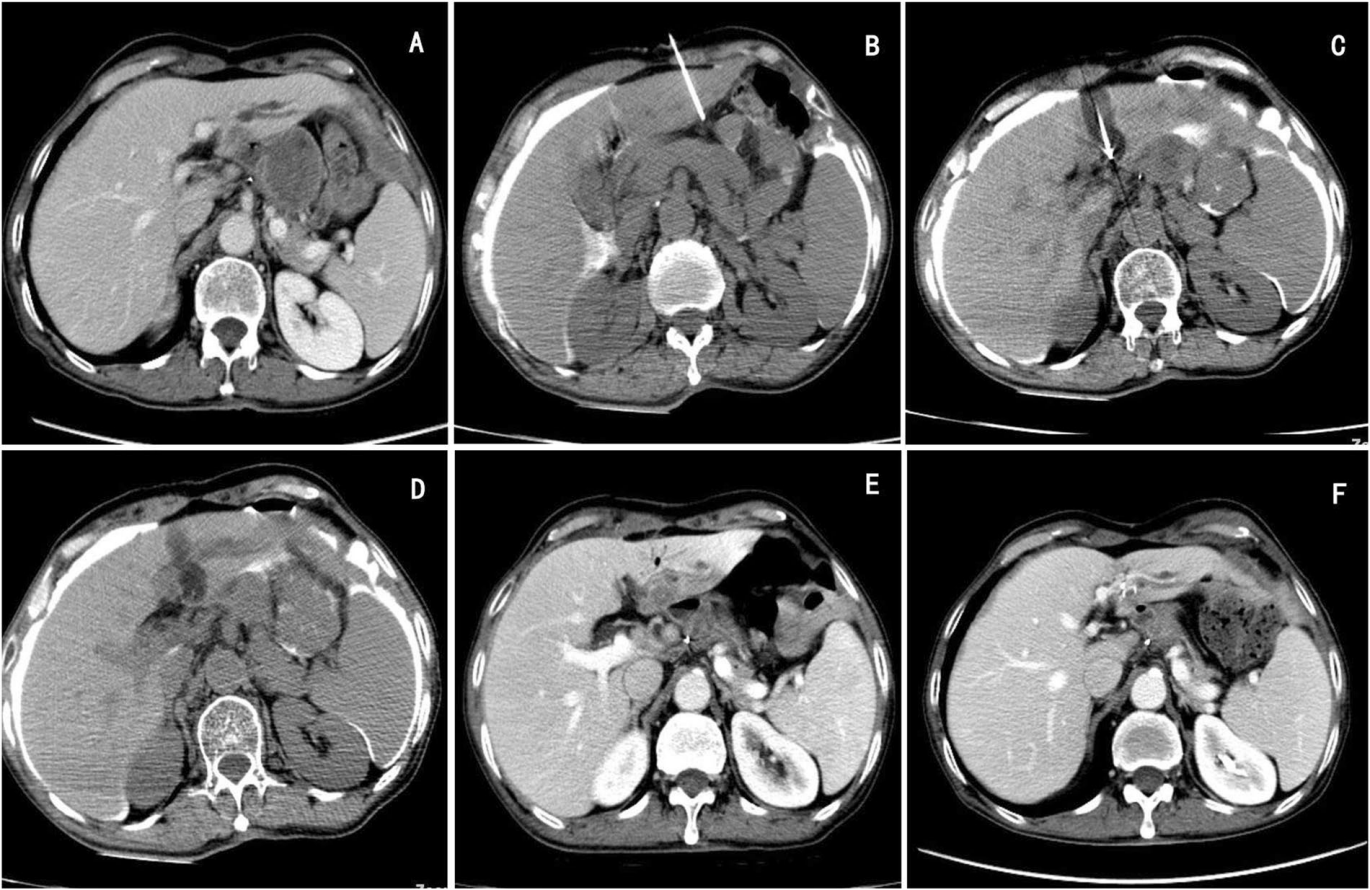
Esophageal carcinoma with liver metastases in a 59 year old man. (**A**) Contrast-enhanced CT demonstrated a low density nodule adjacent to portal vein in left lobe of liver, and there is not clear limit between the nodule and the posterior gastrointestinal tract. (**B**) A 20-gauge introducer needle was inserted into the hepatogastric space under CT guidance, and then a sufficient amount of artificial ascites was injected. (**C**) and (**D**) Cryoablation was applied under CT guidance. (**E**) Contrast-enhanced CT showed no injury in the adjacent gastrointestinal tract after three days follow-up. (**F**) Contrast-enhanced CT showed the focus was shrunk with no signs of recurrence after three months followed up.

Cryoablation was successfully performed in all the patients of two groups under CT-guided procedures. Ablation time of Group - I and Group - II were 12.0 ± 6.0 min (range 12.0–24.0 min) and 12.0 ± 8.0 min (12.0–24.0 min). Maximum diameter of the ablation zone of the two groups was 4.4 ± 1.6 cm (3.3–5.2 cm) and 4.8 ± 2.1 cm (3.3–6.3 cm). Technique effectiveness of Group - I was achieved in 35 of 43 tumors (81.3%) after follow-up imaging three months after cryoablation. In Group - II, technique effectiveness was achieved in 39 of 41 tumors (95%) after follow-up imaging three months later (Figs 1 and 2). There was a statistically significant difference in outcome between two groups. Six cases of Group - I and two cases of Group - II with residual tumor were successfully treated with additional cryoablation, and on follow-up, no further local progression was observed. One patient in Group - I was lost.

**Figure 3:**
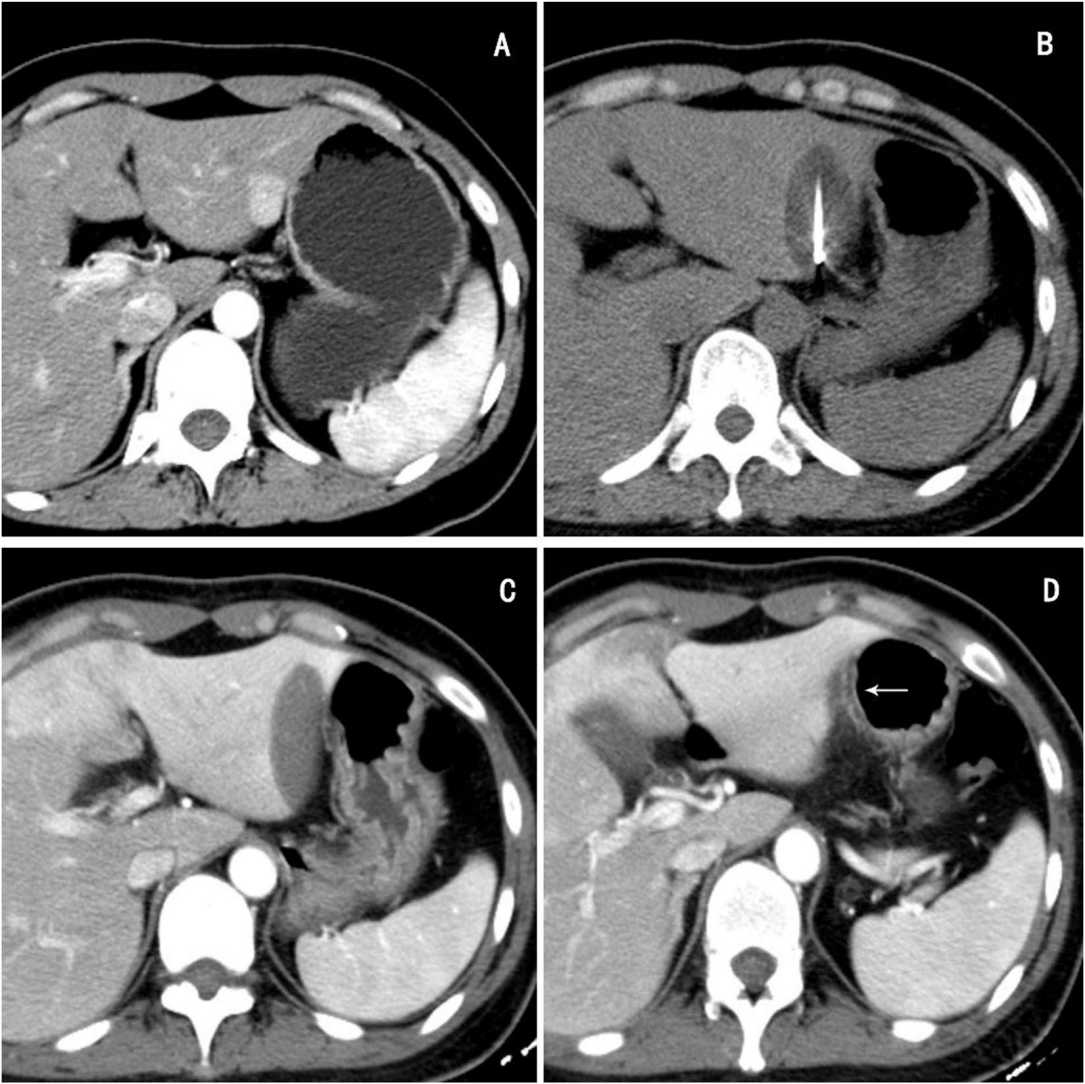
A 34 year old female; (**A**) A nodule in left lobe was detected accidentally during a routine check-up. (**B**) Cryoablation was applied in liver lesion under CT guidance without induction of artificial ascites. (**C**) and (**D**) The CT re-examination within three days showed complete ablation of the lesion (**C**) slight thickening, and edema of the gastric wall adjacent to the lesion (**D**).

No major complications, such as death or cryoshock (which includes fever, multi-organ failure, severe coagulopathy, and disseminated intravascular coagulation), abdominal hemorrhage, hepatic abscess, biliary fistula, or myoglobinuria were encountered in the two groups. After a three month follow-up, six GI injuries (five patients with colonic injury and one patient with stomach injury) was observed in Group - I (Fig. 3), but no GI perforation occurred in either of the two groups. The minor adverse effects of cryoablation are shown in Table 1. Five patients in Group - I and four patients in Group - II were observed with Post Cryoablation Syndrome, which was presented as a syndrome of low-grade fever and general malaise. Right pleural effusion was observed in four patients of the two groups whose tumors were located near the top of the diaphragm. Patients with these complications healed after conservative management. No symptomatic bleeding was seen in either group.

## Discussion

Percutaneous cryoablation can cause cryoinjury to adjacent structures leading to devastating consequences^11^. As the ice ball must extend at least 3.0 mm beyond the outer tumor margin to result in complete cell death, the anatomy of the GI tract in reference to the tumor is important to avoid injury to the structures adjacent to the tumor^5^. Damage to the hollow, thin-walled structures, such as the bowel and ureter, entails more severe complications, like mural necrosis and secondary abscess or stricture, as compare to damage to solid organs^3^.

Many cases of hepatic metastases and hepatocellular carcinoma are peripherally located in close proximity to the GI tract. Artificial ascites act as an insulator between liver and surrounding hollow organs separating them from each other, and as the distance between site of cryoablation and these organs increases, the transmission of cryoinjury to these organ is avoided^12^.

Hence, here in the present study, we have tried to evaluate the protective effect of artificial ascites against injury during cryoablation and the efficiency of tumors cryoablation. As our results have shown, artificial ascites not only can reduce GI tract injury, but it also can increase the ablation effect.

Both human and animal studies have found that the use of peritoneal fluid decreases both collateral injuries and post procedure pain for radio frequency ablation of peripheral liver tumors^13,14^. Asvadi *et al*.^13^ described using microwave ablation treatment for tumors in the hepatic dome. In their study thirty-four tumors (70%) were treated following creation of artificial ascites with 0.9% normal saline solution. The technical success rate was 100%, and the complete response rate was 94%. The overall survival rate was 73.9% over 24.7 months of follow-up with no major complications.

The mediums of artificial ascites were not same. In contrast to majority of the study, where normal saline was used, Sutchin *et al*.^3^ used iodinated contrast solution for artificial induction of ascites before percutaneous renal cryoablation. Moreover, De Benedectis *et al*.^15^ used a mixture of 5.0% dextrose in water and iodinated contrast medium for hydro dissection and found no adjacent structure injury during percutaneous radio frequency ablation of hepatic tumors adjacent to the GI tract. On the third week post intervention follow-up, no residual hydro dissection fluid was noted on the CT scan.

Campbell *et al*.^16^ reported an isohexol concentration of 1:50 to be optimal to produce sufficient contrast between abdominal organs and infused fluid without any contrast and related artifacts such as beam hardening artifacts. Thus, here in the present study we opted to use 2.0% iodinated contrast medium. We observed that attenuation of HU of the hydro dissection fluid varied with its depletion in the abdomen. Moreover it improved the tumor visibility by taking place of the surrounding air in the abdomen.

The mean displacement distance of adjacent structures was 1.3 cm (range 0.5–2.2 cm), which was in agreement with other studies (range 1.6–2.6 cm)^3,15^.

Furthermore, ablation effect was found to be increased with the induction of artificial ascites. The separation success rate in our study was 95% (39/41), and the technical effectiveness of cryoablation in Group - II was significantly higher than it was in Group - I. Adequate ablative margins were achieved with the induction of artificial ascites thereby separating tumors and the GI tract. This in turn would protect the adjacent GI tract from cryoinjury. Alexandra *et al*.^1^ achieved 90% (19/21) of technical success in treating hepatic tumor adjacent to the gallbladder with CT or MRI guided cryoablation. However, two tumors in the portion of the ablation zone that directly abutted the gallbladder recurred and required a second ablation.

Ascites can wash away coagulation substances at the puncture site and cause hemorrhage. It also releases the compression effect of the opposing abdominal wall and organs against liver, facilitating the dissemination of tumor cells. However, in the present study, no tumor seeding or intra peritoneal hemorrhage was observed after cryoablation ablation during follow-up^12^. We believe that during cryoprobes withdrawal, cauterizing the needle track would have prevented these complications in the present study^12^. In our study, no diuretics or paracentesis was used, and residual ascites disappeared spontaneously which was the same as in most previous studies^3,10,12,15^.

Our study was limited by its small sample size which could have conditioned result reported and thus needs a confirmation in a largest trial. Additionally, this study with its retrospective nature had all of the inherent limitations. Another limitation was the relatively short duration of the follow-up. Although we think that the mean imaging follow-up time in this study (≈one year) was sufficient to detect most significant GI tract injuries.

In conclusion, our study shows that use of artificial ascites is a reliable and effective method for the cryoablation ablation of hepatic tumors which are adjacent to the GI tract, and this method can achieve a fine local control of such tumors.

## Methods

### Patients

Our study was approved by the Institutional Review Board of the Affiliated Hospital of North Sichuan Medical College. Informed consent was obtained from all of the patients. All methods of the experiments were performed in accordance with the relevant guidelines and regulations. From Dec. 2014 to Dec. 2016, 79 patients with 84 hepatic tumors (48 hepatocellular carcinomas, 33 metastases, and three hepatocellular adenomas) were treated with CT-guided percutaneous cryoablation at the hospital. The patients inclusion criteria were as follows: hepatic tumors which were not resectionable or in which the patient refuses to undergo surgical resection; an isolated tumor ≤ 5.0 cm or a tumor number ≤ four, with each tumor maximum diameter ≤ 3.0 cm; Liver cirrhosis Child-Pugh class A or B; a prothrombin time < 25 s; and a platelet count > 40 cells × 109/l. All tumors were considered as peripheral when the external boarder of the tumor was located within 0.5 cm from the GI tract. Among the treated patients, 43 peripheral hepatic tumors in 40 patients (26 men and 14 women) were treated with cryoablation without artificial ascites (Group - I), 41 peripheral hepatic tumor in 39 patients (24 men and 15 women) were treated with cryoablation with artificial ascites (Group - II). Table 1 summaries the patients and tumors clinical features at the time of cryoablation.

### Artificial ascites and cryoablation Procedure

After the management of local anesthesia with 2.0% lidocaine to the skin, abdominal wall and peritoneum, a 15.0 cm, 20-gauge introducer needle (Bard, US) was inserted into the peritoneal cavity under CT guidance (Phlips MX16) along the edge of the liver (Fig. 1B). When the needle was inserted into the correct site under CT scan, the inner stylet was removed. Then, a sufficient amount of 2.0% iodinated contrast (Nycomed Ireland Ltd.) was injected until at least 0.5 cm separation between the target tumor and the adjacent GI tract was achieved (Fig. 1C). An argon-based cryoablation delivery system (AccuTarget, China, Shanghai) was used with 17-gauge cryoprobes. The freezing temperature of lethal ice can reach 135.0–150.0 degrees below zero. Depending on the tumors size and location, one to three (mean, 1.5) cryoprobes were used with the goal to include a 5.0–10.0 mm margin of ice ball beyond the tumor. Two freeze/thaw cycles (12.0 min freeze, 3.0 min passive thaw) were applied conventional (Fig. 1C). Sometimes, because the patients had smaller lesions, the CT scan was performed when the cryoablation proceeded at 6.0 min. Once the CT indicated that the ice ball had completely covered the tumor margin more than 3.0 mm, we stopped the cryoablation process to protect the normal structure adjacent to the tumors from injury. Ablation time and power were recorded during all procedures. All patients underwent an immediate post ablation contrast-enhanced CT scan. All patients were admitted for overnight observation. To prevent renal failure, which induced by myoglobinuria, a prophylactic regimen consisting of three ampules of sodium bicarbonate (50.0 mEq per ampule) in 5.0% dextrose in water at 150.0 ml/h for 24 hours was administered if the post procedural serum myoglobin level increased to more than 1,000 mg/L^17^.

### Patient Follow-Up

One month after the ablation, contrast-enhanced CT or MRI was performed to evaluate technique and ablation effectiveness. Complete ablation was defined as: the whole tumor lack of enhancement. If complete ablation was achieved, routine contrast-enhanced CT or MRI was repeated at three months after cryoablation to assess local tumor progression, and then at six month intervals. Progression of local tumor was defined as tumor enhancement reappearance adjacent or within to the ablation zone. Procedure complications were followed up after a one month following cryoablation and detected by using CT, MRI, or ultrasound.

### Data analysis

The control group was designated as cases which no fluid was used. The patient’s age, tumor size, displacement distance/artificial ascites thickness, length of follow-up, power, and time of study treatment cycles were compared statistically by using an unpaired Student’s T-test and Fisher’s Exact Test of SPSS software (version 19.0, SPSS, Chicago, IL). *P* values more than 0.05 were considered statistically significant.

## References

[1] Fairchild AH (2014). Percutaneous cryoablation of hepatic tumors adjacent to the gallbladder: assessment of safety and effectiveness. J Vasc Interv Radiol..

[2] Littrup PJ (2016). Percutaneous cryoablation of hepatic tumors: long-term experience of a large U.S. series. Abdom Radiol (NY)..

[3] Patel SR (2012). Hydro dissection using an iodinated contrast medium during percutaneous renal cryoablation. J Endourol..

[4] Atwell TD (2012). Complications following 573 percutaneous renal radio frequency and cryoablation procedures. J Vasc Interv Radiol..

[5] Lee JM (2015). Risk factors of organ failure in cholangitis with bacteriobilia. World J Gastroenterol..

[6] Chu MW, Wilson SR, Novick RJ, Stitt LW, Quantz MA (2004). Does clopidogrel increase blood loss following coronary artery bypass surgery?. Ann Thorac Surg..

[7] Kim JW (2015). Ultrasound-guided percutaneous radio frequency ablation of liver tumors: how we do it safely and completely. Korean J Radiol..

[8] Kitchin D (2014). Microwave ablation of malignant hepatic tumors: intra peritoneal fluid instillation prevents collateral damage and allows more aggressive case selection. Int J Hyperthermia..

[9] Iwai S (2012). Benefits of artificially induced pleural effusion and/or ascites for percutaneous radio frequency ablation of hepatocellular carcinoma located on the liver surface and in the hepatic dome. Hepatogastroenterology..

[10] Nam SY (2010). Percutaneous radio frequency ablation for hepatic tumors abutting the diaphragm: clinical assessment of the heat-sink effect of artificial ascites. AJR Am J Roentgenol..

[11] Tsivian M (2010). Complications of laparoscopic and percutaneous renal cryoablation in a single tertiary referral center. Eur Urol..

[12] Zhang M (2014). Efficacy and safety of artificial ascites in assisting percutaneous microwave ablation of hepatic tumors adjacent to the gastrointestinal tract. Int J Hyperthermia..

[13] Asvadi, N. H. *et al*. CT-guided percutaneous microwave ablation of tumors in the hepatic dome: assessment of efficacy and safety. *J Vasc Interv Radiol*. **27**, 496–502, 503 (2016). 10.1016/j.jvir.2016.01.010 26922977

[14] Fu JJ (2016). Effect of thermo-sensitive matrigel on minimization of thermal injury to the nearby structures in radio frequency ablation of subcapsular hepatic tumors in a Rat Model. Zhonghua Yi Xue Za Zhi..

[15] DeBenedectis CM, Beland MD, Dupuy DE, Mayo-Smith WW (2010). Utility of iodinated contrast medium in hydro dissection fluid when performing renal tumor ablation. J Vasc Interv Radiol..

[16] Campbell C, Lubner MG, Hinshaw JL, Munoz DRA, Brace CL (2012). Contrast media-doped hydro dissection during thermal ablation: optimizing contrast media concentration for improved visibility on CT Images. AJR Am J Roentgenol..

[17] Nair RT (2008). Biochemical and hematologic alterations following percutaneous cryoablation of liver tumors: experience in 48 procedures. Radiology..

